# Properties of Manganese(III) Ferrocenyl-β-Diketonato Complexes Revealed by Charge Transfer and Multiplet Splitting in the Mn 2p and Fe 2p X-Ray Photoelectron Envelopes

**DOI:** 10.3390/molecules21111427

**Published:** 2016-10-26

**Authors:** Blenerhassitt E. Buitendach, Elizabeth Erasmus, J. W. (Hans) Niemantsverdriet, Jannie C. Swarts

**Affiliations:** 1 Department of Chemistry, University of the Free State, Bloemfontein 9300, South Africa; blenerbuitendach@gmail.com (B.E.B.); ErasmusE@ufs.ac.za (E.E.); 2 SynCat@DIFFER, Syngaschem BV, De Zaale 20, Eindhoven 5612 AJ, The Netherlands; j.w.niemantsverdriet@tue.nl

**Keywords:** manganese, ferrocene, β-diketonato complexes, multiplet splitting, charge transfer, X-ray photoelectron spectroscopy

## Abstract

A series of ferrocenyl-functionalized β-diketonato manganese(III) complexes, [Mn(FcCOCHCOR)_3_] with R = CF_3_, CH_3_, Ph (phenyl) and Fc (ferrocenyl) was subjected to a systematic XPS study of the Mn 2p_3/2_ and Fe 2p_3/2_ core-level photoelectron lines and their satellite structures. A charge-transfer process from the β-diketonato ligand to the Mn(III) metal center is responsible for the prominent shake-up satellite peaks of the Mn 2p photoelectron lines and the shake-down satellite peaks of the Fe 2p photoelectron lines. Multiplet splitting simulations of the photoelectron lines of the Mn(III) center of [Mn(FcCOCHCOR)_3_] resemble the calculated Mn 2p_3/2_ envelope of Mn^3+^ ions well, indicating the Mn(III) centers are in the high spin state. XPS spectra of complexes with unsymmetrical β-diketonato ligands (i.e., R not Fc) were described with two sets of multiplet splitting peaks representing *fac* and the more stable *mer* isomers respectively. Stronger electron-donating ligands stabilize *fac* more than *mer* isomers. The sum of group electronegativities, Σχ_R_, of the β-diketonato pendant side groups influences the binding energies of the multiplet splitting and charge transfer peaks in both Mn and Fe 2p_3/2_ photoelectron lines, the ratio of satellite to main peak intensities, and the degree of covalence of the Mn–O bond.

## 1. Introduction

Properties of metal(III) β-diketonato complexes [M(R^1^COCHCOR^2^)_3_], M = metal and R = pendant β-diketonato side groups such as CH_3_, have been studied with a variety of different techniques including crystallography [1], electrochemistry [2], non-linear refractive measurements [3], UV-Vis spectroscopy [4], and high frequency electron paramagnetic resonance (EPR) [5]. However, characterization of these complexes by means of X-ray photoelectron spectroscopy (XPS) is not well established. XPS can play an important role in further determining the influence of the chemical environment surrounding the elements under investigation. The binding energy of a photoelectron line generally gives insight into the oxidation state of the metal ion. However, the position and shape of the photoelectron line is further influenced by final-state effects of the metal ions. These final state effects include multiplet splitting, shake-up, and shake-down peaks which are caused by crystal field splitting and charge transfer from the ligand to the metal (back bonding) [6,7]. The substructure obtained due to Mn 2p (2p^5^3d*^n^*^+1^) final state effect can be traced back to the 3d core-level and the valence band of the manganese. For octahedral structures such as Mn(β-diketonato)_3_ complexes, the crystal field splits the 3d level into a higher energy doubly degenerate (*e_g_*) level and a lower energy triply degenerate (*t_2g_*) level. The crystal potential determines the difference between the *e_g_* level and *t_2g_* level, and this difference is large for octahedral complexes. If the interaction between the unpaired core p-electrons (2p core holes) and the unpaired valence 3d-electrons are strong enough, satellite shake-up peaks which result from valence electrons being transferred to unoccupied states are present in the XPS spectra at a few eV higher than the main photoelectron lines.

Multiplet splitting of photoelectron lines arises from the coupling of the angular momenta (spin-orbit coupling) of the unpaired core p-electrons (caused by photoionization) and the unpaired valence d-electrons [8,9]. Paramagnetic high-spin metal species show significant multiplet splitting [10]. The full width at half maximum (FWHM) of the photoelectron lines of these high-spin metals are broader than those of their low-spin counterparts—e.g., high spin Mn(III) vs. low spin Mn(II) [5]. These complex photoelectron lines can be deconvoluted with multiplet splitting peaks as demonstrated by Gupta and Sen [8,9] and others [11,12,13].

In the XPS of complexes bearing 3d transition metals there normally appear shake-up peaks at a few eV higher than the main peak’s binding energy. This is generally accepted to be the charge transfer peak from the ligand orbital to the metal *3d*, *4s*, or *4p* orbitals [14,15,16,17,18,19].

Multiplet splitting in XPS spectra of metal oxides and metal hydroxides has been investigated intensively [10,13,20,21,22]. There also are reports on multiplet splitting of metal halides, phosphates, and metal sulphides [13,22]. It has, however, never been attempted to deconvolute the multiplet splitting of Mn β-diketonates and relate the peaks to the free ion splitting as calculated by Gupta and Sen [8,9].

**Figure 1:**
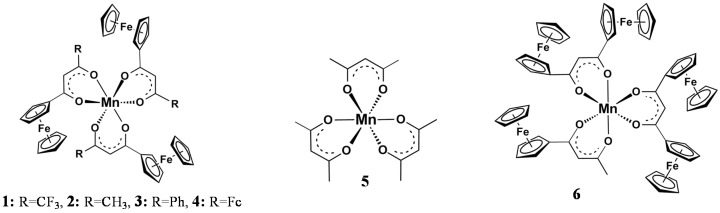
Structures of **1**–**6**.

Useful mathematical relationships exist between the Gordy group electronegativity [23], χ_R_, of different pendent R-groups of the β-diketonato ligands, (R^1^COCHCOR^2^)^−^ and other physical properties including NMR resonance positions, redox potentials, reaction rates, and ligand pK_a_’s [24,25,26,27,28,29,30,31]. With respect to XPS determined binding energies we recently reported [4] that for [Mn(FcCOCHCOR)_3_] complexes with R = CF_3_, **1**, CH_3_, **2**, C_6_H_5_, **3**, and Fc, **4**, as well as [Mn(CH_3_COCHCOCH_3_)_3_], **5**, and [Mn(FcCOCHCOFc)_2_(FcCOCHCOCH_3_)], **6**, (Figure 1) linear relationships exist between the binding energies at maximum peak height of the Fe 2p_3/2_ and Mn 2p_3/2_ photoelectron lines and R-group’s Gordy scale group electronegativities, χ_R_, as well as Σχ_R_ = χ_R1_ + χ_R2_ where R^1^ and R^2^ are the group electronegativities of pendant side groups of the β-diketonato ligands (R^1^COCHCOR^2^)^−^ [4]. However, χ_R_, and Σχ_R_ may also relate to the substructures (shake-up, shake-down, and multiplet splitting) of the Mn 2p_3/2_ and Fe 2p_3/2_ photoelectron lines [32].

Here, we report results from an in depth analysis of the (a) satellite substructure as well as (b) multiplet splitting substructure of the manganese(III) and ferrocenyl iron(II) metal centers and empirically quantify these substructure binding energies as a function of χ_R_ and Σχ_R_ of the ligand R-groups.

## 2. Results and Discussion

**Figure 2:**
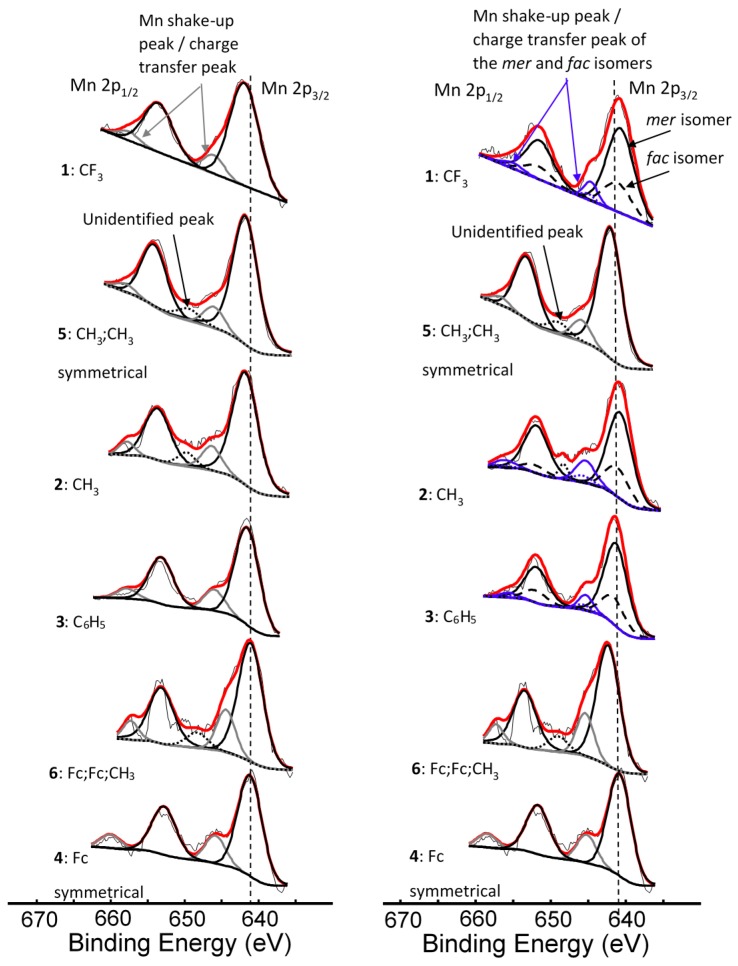
**Left**: Comparative XPS spectra showing a single peak fitted for all the main Mn 2p peaks as well as the shake-up peaks of the Mn 2p area of complexes **1**–**4**, [Mn(FcCOCHCOR)_3_], **5**, [Mn(CH_3_COCHCOCH_3_)_3_] and **6**, [Mn(FcCOCHCOFc)_2_(FcCOCHCOCH_3_)]. **Right**: Comparative XPS spectra showing one peak fitted for the main Mn 2p peaks of the complexes having symmetrical β-diketonato ligands and two simulated peaks for the complexes with unsymmetrical β-diketonato ligands, representing the *mer* and *fac* isomers (a ratio of 3:1 was forced into the simulation) as well as the shake-up peaks of the Mn 2p area of complexes **1**–**6**. In some cases, a surface peak from the interface of Mn complexes attached to the carbon tape was identified and is shown with ^….^ lines. The vertical dotted lines give an indication of how the binding energy shifts.

XPS data of the β-diketonato manganese(III) complexes **1**–**6** (Figure 1) with respect to the Mn 2p and Fe 2p regions are presented in Table 1, Table 2 and Table 3. The broad peaks of the Mn 2p_3/2_ and Mn 2p_1/2_ photoelectron lines (a wide envelope) are located between 641.31–641.86 eV and 652.82–653.56 eV respectively (Figure 2, Table 1).

The binding energy position of the binding energies at maximum peak height of the main Mn 2p_3/2_ photoelectron line corresponds to Mn^III^ [33]. It is known that Mn^III^ exists in a high-spin paramagnetic state [34,35], which also is evident from the high FWHM (ca. 3.86–4.64 eV). A strong satellite peak at ca. 4.4 eV higher than the maximum binding energy of the Mn 2p photoelectron lines is also present (Figure 2). An additional unidentified peak at ca. 8 eV higher than the maximum binding energy of the Mn 2p_3/2_ photoelectron line (prominently observed for **2**, **3**, **4**, and **6**, and to a lesser extent for **1** and **5**) was observed.

**Figure 3:**
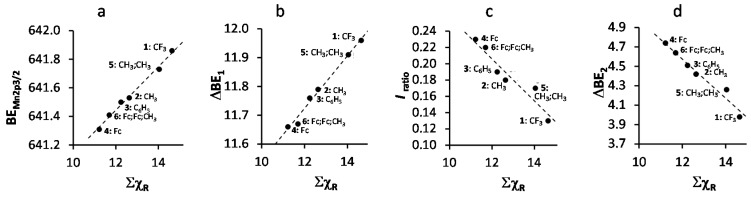
(**a**) Relationship between the binding energy of the main Mn 2p_3/2_ photoelectron envelope and the sum of β-diketonato ligand Gordy scale R-group electronegativities, Σχ_R_, of **1**–**6**; (**b**) Relationship between the spin orbit splitting of the main Mn 2p_3/2_ and Mn 2p_1/2_ photoelectron envelopes (ΔBE_1_ = BE_Mn2p1/2_ − BE_Mn2p3/2_) and Σχ_R_; (**c**) Relationship between the ratio of the intensities of the satellite and main Mn 2p_3/2_ photoelectron line, *I_ratio_* *=* (*I*_Mn2p3/2satel_)/(*I*_Mn2p3/2main_), and Σχ_R_ of **1**–**6**; (**d**) Relationship between the difference between the maximum binding energy of the main Mn 2p_3/2_ photoelectron line and the satellite Mn 2p_3/2_ photoelectron line (ΔBE_2_ = BE_Mn2p3/2satel_ − BE_Mn2p3/2main_) and Σχ_R_ of **1**–**6**.

A spin orbit splitting of 11.58–11.96 eV was observed, depending on Σχ_R_ of the R-group on the β-diketonato ligand. An increase in Σχ_R_ leads to an increase in binding energy (Figure 3a, we discussed this relationship in detail before) and the spin orbit splitting between the Mn 2p_3/2_ and Mn 2p_1/2_ photoelectron lines (Figure 3b). The latter relationship is an indication of dependence on the degree of delocalization of the spin density on the valence orbitals [36,37].

In paramagnetic compounds, the intensity of the satellite and main peaks are important. The ratio of the intensities of the satellite and main Mn 2p_3/2_ photoelectron lines, *I_ratio_* = (*I*_Mn2p3/2satel_)/(*I*_Mn2p3/2main_), correlates with spin density (magnetic moment, i.e., increasing number of unpaired electrons) [38,39]. Here we demonstrate that *I_ratio_* (and therefore also spin density) also correlates to Σχ_R_, Figure 3c. To interpret this relationship, we note the amount of unpaired electrons in all Mn(III) complexes **1**–**6** are the same. This implies the effect of the increase in polarity (electron density shift to one side of the β-diketonato ligand) of the valence electrons due to increased Σχ_R_ could be considered proportional to the increased magnetic moment or spin density. Therefore, there must be a correlation between the spin density and the electronegativity of the ligand. From Figure 3c (and values for *I_ratio_* in Table 1) it is evident that the lower Σχ_R_ is (Σχ_Fc_ = 11.22 for **4** is the smallest), the larger the satellite peak gets.

It is known that observed satellite peaks for first row transition metal complexes with an octahedral symmetry is attributed to charge transfer from ligand-to-metal transitions of the type *e_g_*-e_g_^*^ and/or *t_2g_*-t_2g_^*^ [40]. A donor-acceptor bond is formed, which transfers electron density from the ligand to the metal center via a σ-donor mechanism. A larger satellite charge transfer peak indicates that more charge (electron density) is transferred from the ligand to the metal. The graph in Figure 3c confirms this. Since β-diketonato ligands with lower Σχ_R_ values, e.g., Σχ_Fc_ = 11.22 for **4** which supports in total 6 strongly electron-donating ferrocenyl groups in its structure, are stronger electron-donating than others, they would transfer more charge to the Mn metal center than the ligands of, for example, **1** with three Fc and three CF_3_ groups and Σχ_R_ = 14.64. Thus, our obtained relationship between Σχ_R_ and *I_ratio_* is consistent with the argument that more electron-donating ligands would transfer more charge to the metal center they are coordinated to than weaker electron-donating ligands.

The molecular orbital energy of a Mn-ligand bond depends on the overlap of the Mn 4s orbitals and ligand orbitals. Larger overlap of metal and ligand orbitals will lead to a lower energy of the bonding orbital and a higher energy of the anti-bonding orbital. Since the ligand orbitals have a lower energy than the 4s orbital of the metal center, the bonding orbital will predominantly be located over the ligand orbital, while the antibonding orbital will then mainly be located over the metal center 4s orbital. The satellite XPS peak represents the charge-transfer process from the ligand 3d orbital to the metal 4s orbital. Therefore, the difference between the maximum binding energy of the satellite Mn 2p_3/2_ photoelectron line and the main Mn 2p_3/2_ photoelectron line, ΔBE_2_ = BE_Mn2p3/2satel_ − BE_Mn2p3/2main_ (values are summarized in Table 1), can be used as a measure of the degree of covalence of the ligand-metal bond. From Figure 3d, an increase Σχ_R_ of the complexes **1**–**6** leads to a decrease in ΔBE_2_. This implies that the degree of the difference in covalency between the initial and final states of the ligand-metal bond decreases as Σχ_R_ increases. It follows that the Mn–O bond of **1** with R = CF_3_ has a considerably lower degree of difference in covalency between the initial and final states than **4** with R = Fc. This is in agreement with what was found [41] for a series of Mn-halides, where MnF_2_ has a lower covalency than MnBr_2_ (Br has a lower Pauling electronegativity [42], χ_Br_ = 2.96, than F; χ_F_ = 4.00).

The linear relationship obtained between spin orbit splitting of the Mn 2p_3/2_ and Mn 2p_1/2_ photoelectron lines (ΔBE_1_), the ratio of the intensities of the satellite and main Mn 2p_3/2_ photoelectron line (*I*_ratio_), and the difference between the maximum binding energy of the satellite Mn 2p_3/2_ photoelectron line and the main Mn 2p_3/2_ photoelectron line (ΔBE_2_) on the one hand and the sum of β-diketonato ligand Gordy scale R-group electronegativities, Σχ_R_ (Figure 3) on the other fit the equations:

BE_Mn2p3/2_ = 0.1517 Σχ_R_ + 639.62; R^2^ = 0.9919 (1)

ΔBE_1_ = BE_Mn2p1/2_ − BE_Mn2p3/2_ = 0.0911 Σχ_R_ + 10.631; R^2^ = 0.9867 (2)

*I_ratio_* = (*I*_Mn2p3/2satel_)/(*I*_Mn2p3/2main_) = −0.0258 Σχ_R_ + 0.5148; R^2^ = 0.9111 (3)

ΔBE_2_ = BE_Mn2p3/2satel_ − BE_Mn2p3/2main_ = −0.2013 Σχ_R_ + 6.99; R^2^ = 0.9621 (4)

The above relationships enable one to calculate (or predict) the degree of delocalization of electrons, amount of charge transferred via a σ-donor mechanism, as well as the degree of the difference in covalency between the initial and final states for manganese(III) β-diketonato complexes **1**–**6** as well as related complexes provided the group electronegativity of β-diketonato R-group substituents are known.

**Figure 4:**
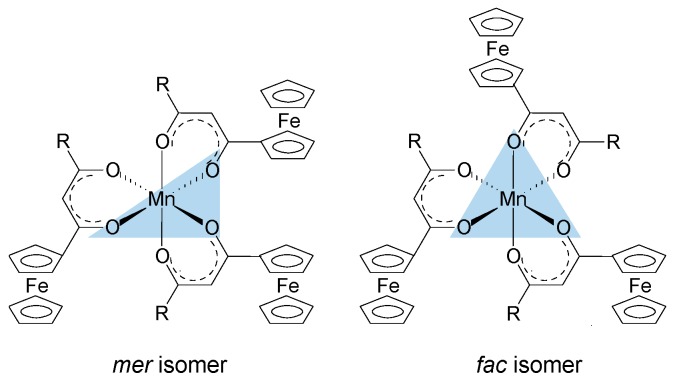
*Mer* and *fac* isomers of [Mn(FcCOCHCOR)_3_] complexes containing an unsymmetrically substituted β-diketonato ligand, R = CF_3_ (**1**), CH_3_ (**2**), C_6_H_5_ (**3**).

The oxygen atoms of the ferrocenyl-functionalized β-diketonato manganese(III) complexes, **1**–**4** and **6**, as well as the *tris*(acetylacetonato)manganese(III) complexes **5** are octahedrally arranged around the Mn metal center. When the Mn(III) complexes contain symmetrically substituted β-diketonato ligands, as in **4** and **5**, the six Mn–O bonds are equal and these complexes display a *D*_3_ symmetry. However, when the Mn(III) complexes contain unsymmetrically substituted β-diketonato ligands, as in **1**–**3**, *mer* and *fac* geometrical isomers are possible (Figure 4). The meridional isomers (*mer*) possess a symmetry plane passing through the Mn metal center which is formed by the three identical substituents (in this case the ferrocenyl groups) on the three unsymmetrical ligands. For the facial isomer (*fac*), the vertices of one face of the octahedron of the Mn complexes are occupied by the same substituent (in this case the ferrocenyl groups) from the three unsymmetrical ligands.

For the three complexes **1**–**3** having unsymmetrically substituted β-diketonato ligands, two Gaussian peaks were simulated to the Mn main 2p_3/2_ peak as well as to the shake-up peak; one was associated with the *mer* and the other with the *fac* isomer. Since the distribution between *mer* and *fac* isomers statistically should be 3:1 for all three complexes, the two Gaussian peaks were enforced in a ratio of 3:1. This simulation with two Gaussian peaks (FWHM = 3.90, 3.68, 3.68 eV for **1**, **2**, and **3** respectively and CHI squared = ca. 1.3) was more accurate than the fitting of only one Gaussian peak (FWHM = 4.64, 4.00, 3.86 eV for **1**, **2**, and **3** respectively CHI squared = ca. 1.5) and the FWHM decreased. The peak of the *fac* isomer (dashed lines) is found at ca. 0.4 eV higher binding energy than the peak of the *mer* isomer (solid lines), see Figure 2 and Table 1. The higher binding energy of the *fac* isomer is in agreement with published results [29], as well as experimental evidence of a similar Al compound, which showed that in solution only the *mer* isomer is present [43]. This implies that the *mer* isomer is more stable and in XPS the more stable isomer would be detected at the lowest binding energy. The difference in binding energies between *mer* and *fac* isomers has, to our knowledge, not been explained theoretically before. Our XPS measurements, however, shows experimentally they are different. To place this experimental result in perspective, it is useful to note that DFT calculations on a similar set of tris(β-diketonato)manganese(III) complexes revealed there is an energy difference between *mer* and *fac* HOMO energies [44]. Since E_HOMO_ has been proven to be related to binding energies obtained from XPS [30,45,46], it follows that there must also be a difference between the binding energies of the *fac* and *mer* isomers. Qualitatively, this means interaction between different molecular fragments (Fc and R; R and Fc cannot equal in *mer* and *fac* isomers) in different special arrangements relative to each other must induce small but noticeable differences in the binding energies for these *fac* and *mer* isomers.

The difference in binding energy position for the *mer* and *fac* isomer was found to be dependent on Σχ_R_, the sum of the Gordy group electronegativity of the R-groups substituted on the ferrocenyl-containing β-diketonato ligands. From Table 1, it is evident that as Σχ_R_ increases, the difference between the *mer* and the *fac* isomer binding energies also increases. This implies that more electron-donating R-groups like the ferrocenyl moiety (Fc) stabilizes the *fac* isomer more than electron-withdrawing R-groups like CF_3_. The higher stability of the *fac* isomer of more electron-donating R-groups was also found with DFT calculations of similar Cr(III) and Fe(III) β-diketonato complexes [40].

**Figure 5:**
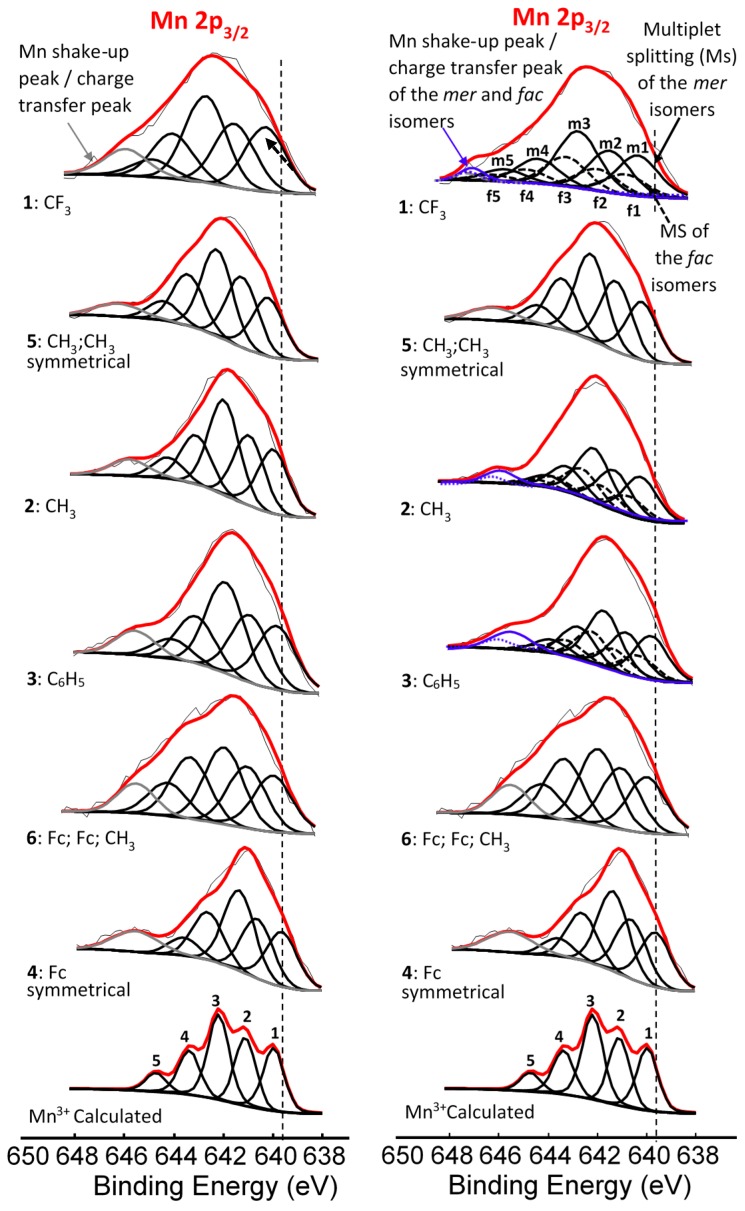
**Left**: Comparative XPS spectra showing muliplet splitting for the main Mn 2p_3/2_ peaks as well as the shake-up peak of the Mn 2p_3/2_ of complexes **1**–**6**. **Right**: Multiplet splitting (Ms) of both the *mer* (solid line) *fac* (dashed line) isomers. In both XPS comparisons, simulated spectra of the Gupta and Sen calculated multiplet splitting of Mn^3+^ is superimposed onto experimental spectra. The vertical dotted lines give an indication of how binding energy shifts.

The Mn(III) atom in complexes **1**–**6** all have an initial state 2p^6^3s^2^3p^6^3d^4^ subshell population. After photoionization, a final state of 2p^5^3s^2^3p^6^3d^4^ subshell population is present [47]. Thus the Mn 2p XPS spectra of complexes **1**–**6** should display a similar multiplet splitting substructure as Gupta and Sen calculated for the core *p*-level of the free Mn^3+^ ion [8,9]. To demonstrate that the experimentally measured Mn 2p_3/2_ photoelectron lines could successfully be generated by the theoretically calculated multiplet splitting peaks of Gupta and Sen, spectra of complexes **1**–**6** were simulated with one set of multiplet split peaks consisting of five components according to the most intense Gupta and Sen calculated peaks for a high-spin Mn^3+^ ion (peaks 1–5, Figure 5, left) in a ratio area % the same as the calculated ratio area distributions of Gupta and Sen for the free Mn^3+^ ion. These were ca. 1:1:1.35:0.7:0.3 [8,9]. Similar successful fittings utilizing the Gupta and Sen distribution were published for manganese oxides, phosphates and sulphates [47,48].

For the three complexes containing symmetrically substituted β-diketonato ligands and two separate types of ligands, complexes **4**–**6**, the single set of five multiplet splitting peaks gave excellent fits, with a CHI squared = ca. 1.2. However, for the complexes containing unsymmetrically substituted β-diketonato ligands, **1**–**3**, where both a *mer* isomer and *fac* isomer are possible, the fitting of two different sets of multiplet splitting peaks (labelled *m1*–*m5* and *f1*–*f5*) into the broad Mn 2p_3/2_ peak (main envelope), proved to be more accurate than the fitting of only one set of multiplet splitting peaks, to give a CHI squared = ca. 0.9 for two peaks as compared to a CHI squared = ca. 1.3 (see Figure 5 right, top).

This is an indication that two different species are present within the sample of complex **1**–**3**. These two different species are attributed to the *mer* and *fac* isomers of the unsymmetrical substituted ferrocenyl-containing β-diketonato manganese(III) complexes. The weight % of the *mer* and *fac* isomers fitted for the XPS data was, as for Figure 2, again 3:1, due to the statistical possibility of occurrence. The XPS data of the multiplet splitting fits of the main envelope of the Mn 2p_3/2_ peaks are listed in Table 2. The multiplet splitting simulations correlated very well with the reported measured data of different manganese oxides, phosphates, and sulphates [47,48].

This implies that, according to the multiplet splitting simulations, the final-state obtained after photoionization of the Mn(III) species is within experimental error independent of the ligand type, even if the ligand varies from an oxide to a phosphates, sulphates, and finally to β-diketonato ligands. The measured fits of the multiplet splitting of the Mn 2p_3/2_ envelopes represent the calculated fittings as predicted by Gupta and Sen well; only the binding energy position differs slightly. This is to be expected since the group electronegativities of the ligands play an important role in the position of the binding energy.

**Figure 6:**
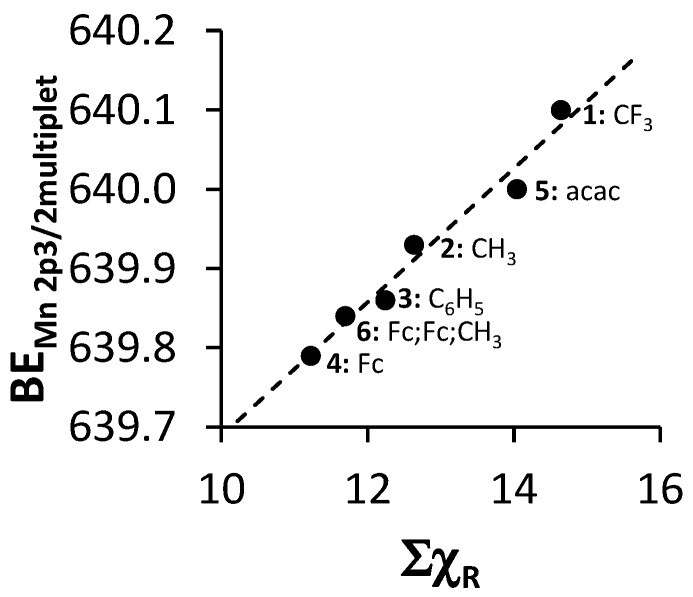
Relationship between the binding energy of the first peak of the *mer* isomers’ fitted multiplet splitting peaks of the Mn 2p_3/2_ photoelectron line of **1**–**3** or the first peak of the fitted multiplet splitting peaks of the Mn 2p_3/2_ phototelectron line of **4**–**6** (BE_Mn2p3/2multiplet_) and Σχ_R_.

The relationship between Σχ_R_ and the binding energy of the first peak of the fitted multiplet splitting peaks of the Mn 2p_3/2_ photoelectron line of **4**–**6** and the first peak of the *mer* isomers’ fitted multiplet splitting peaks of the Mn 2p_3/2_ photoelectron line of **1**–**3** (see Table 2 and Figure 6) was found to be directly proportional. With an increase in Σχ_R_, increased electron density moves away from the Mn^3+^ metal center towards the ligand. The Mn^3+^ ion binds more firmly to its own electrons, causing the increased binding energy values. 

The linear relationship obtained between binding energy of the first peak of the fitted multiplet splitting peaks of the Mn 2p_3/2_ phototelectron line of **4**–**6** and the first peak of the *mer* isomer fitted multiplet splitting peaks of the Mn 2p_3/2_ photoelectron line of **1**–**3** (BE_Mn2p3/2multiplet_) and Σχ_R_ (Figure 6) fits the equation

BE_Mn2p3/2__multiplet_ = 0.0943 Σχ_R_ + 638.85; R^2^ = 0.9691 (5)

**Figure 7:**
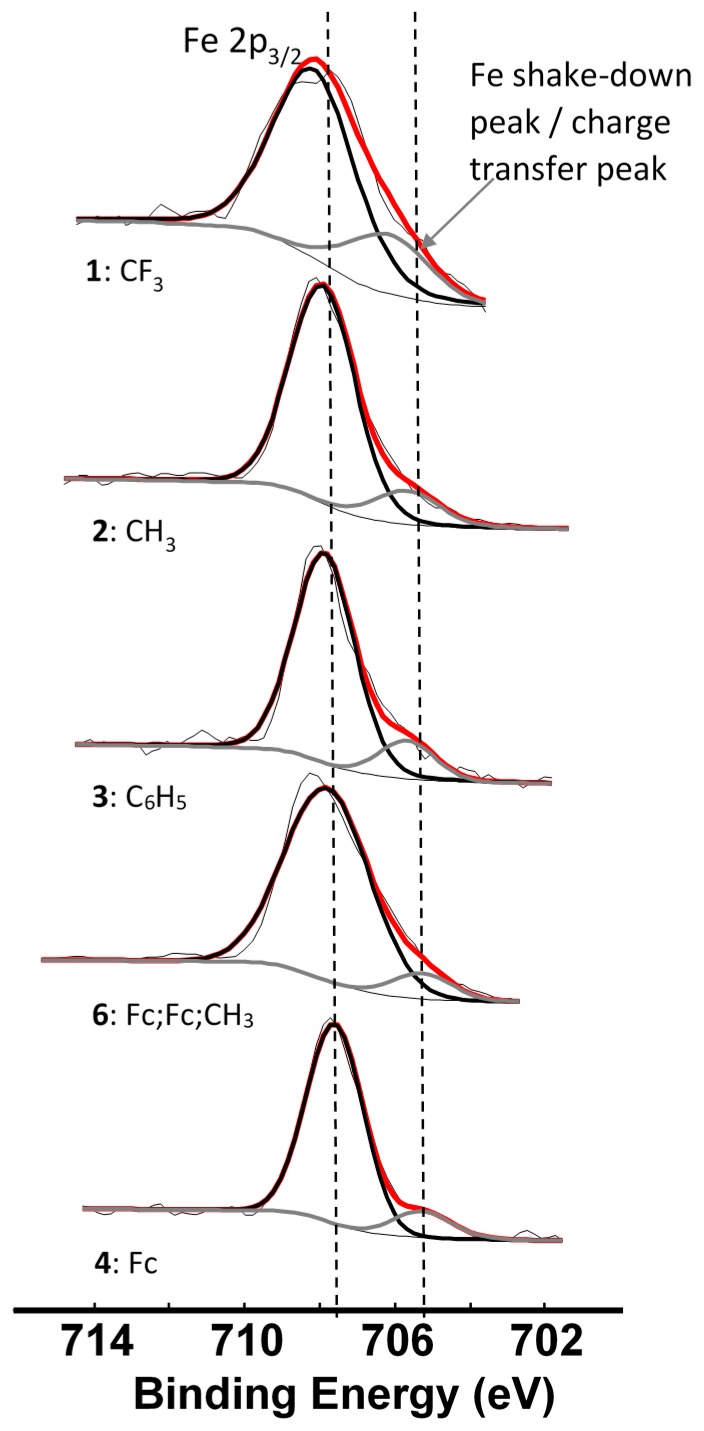
Comparative XPS spectra showing the main Fe 2p_3/2_ peaks as well as the shake-down peaks of complexes **1**–**4** and **6**. The vertical dotted lines give an indication of how binding energy shifts.

Since we could observe the charge transfer process from the β-diketonato ligand to Mn in the Mn 2p spectra, it would only be logical that we should be able to identify a similar charge transfer peak in photoelectron lines representing the ligand. The complexes under investigation are ferrocenyl-containing β-diketonates; therefore the Fe 2p photoelectron line is representative of the ligand. For complexes **1**–**4** and **6**, the Fe 2p_3/2_ photoelectron lines have a maximum binding energy at ca. 708 eV, with a distinct shoulder on the lower energy side of the main photoelectron line, Figure 7. This shoulder is formed by a shake-down mechanism and it represents the charge transfer from the β-diketonato ligand to Mn. The main Fe 2p_3/2_ photoelectron lines are sharp single peaks and since the iron in the ferrocenyl moiety is Fe^2+^, it is in a low spin state and does not display multiplet splitting.

**Figure 8:**
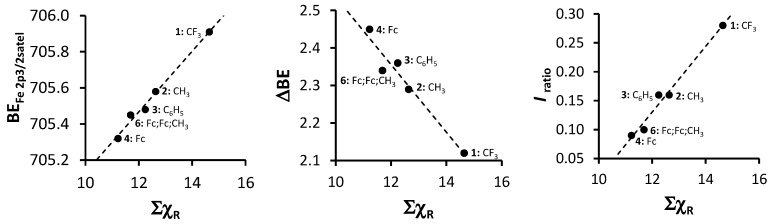
**Left**: Relationship of binding energy of the satellite Fe 2p_3/2_ phototelectron line of **1**–**4** and **6** (BE_Fe2p3/2satel_) and the sum of β-diketonato ligand Gordy scale R-group electronegativities, Σχ_R_. **Middle**: Relationship between ΔBE = BE_Fe2p3/2main_ − BE_Fe2p3/2satel_ and Σχ_R_. **Right**: Relationship between *I_ratio_* = (*I*_Fe2p3/2satel_)/(*I*_Fe2p3/2main_) and Σχ_R_.

An in-depth discussion of the main Fe 2p photoelectron lines of **1**–**4** and **6** were already presented before by us [4], so here only the shake down peak will be discussed. The data obtained from the XPS for the Fe 2p_3/2_ main and satellite peaks are summarized in Table 3. As Σχ_R_ increases, the binding energy of the main Fe 2p_3/2_ photoelectron line also increases. A similar trend was observed for the Fe 2p_3/2_ satellite peaks (Figure 8, left, Table 3). However, the degree of increase in binding energy for the main and satellite Fe 2p_3/2_ peaks are not the same and the difference between the Fe 2p_3/2_ main and the Fe 2p_3/2_ satellite peaks (ΔBE = BE_Fe2p3/2main_ − BE_Fe2p3/2satel_) is inversely proportional to the Σχ_R_ (Figure 8, middle). As Σχ_R_ increases, the difference between the main and the satellite Fe 2p_3/2_ photoelectron lines, ΔBE, decreases.

The charge transfer process takes place between the 3d orbitals of the β-diketonato ligand oxygens and the 4s of the manganese. Thus, when charge is transferred to the manganese, an electron deficiency in the β-diketonato ligand is the result. More specifically, the electron deficiency manifests at the delocalized electrons in the six-membered chelating ring of the coordinated β-diketonato ligand. Stronger electron-donating pendent β-diketonato R-groups (e.g., ferrocenyl) would be able to stabilize this electron deficiency much better than electron-withdrawing R-groups such as CF_3_. This enhanced stabilization would cause the binding energy of the Fe shake-down satellite peak to be located at lower binding energies in comparison to the main Fe 2p_3/2_ photoelectron line, which explains the inverse proportional correlation.

Comparison of the ratio of intensities of the satellite and main Fe 2p_3/2_ photoelectron line (*I_ratio_* = (*I*_Fe2p3/2satel_)/(*I*_Fe2p3/2main_) with Σχ_R_ revealed a direct proportional correlation (Figure 8, right) showing that as Σχ_R_ increases, the intensity of the satellite Fe 2p_3/2_ photoelectron line also increases. This is opposite to the trend found for the intensity ratios of the satellite and main Mn 2p_3/2_ photoelectron line vs. Σχ_R_ (Figure 3c).

After charge transfer from the ligand to the manganese, a partially positive charge, δ^+^, is created on the six-membered chelating ring of the coordinated β-diketonato ligand. When an electron-withdrawing R-group like CF_3_ is attached to this chelating ring (as in complex **1**), it withdraws even more electron density causing an even bigger partial positive charge, δ*^n^*^+^ (1 < *n* < 2). To compensate for this overly δ*^n^*^+^ charge, the electron-donating ferrocenyl groups on the other side of each of the β-diketonato ligands, donates extra electron density to the chelating pseudo-aromatic β-diketonato ring. This causes the intensity of the Fe 2p_3/2_ shake-down peak to be relatively large. When there are two electron-donating ferrocenyl groups on each coordinating β-diketonato ligand, as in **4**, more ferrocenyl groups donate electron-density to compensate for the δ*^n^*^+^ charge. Thus, the individual contributions from each ferrocenyl group (and also the intensity of the charge transfer peak) will be less than for complex **1** bearing CF_3_ electron-withdrawing groups.

The linear relationship obtained between binding energy of the satellite Fe 2p_3/2_ photoelectron line of **1**–**4** and **6** (BE_Fe2p3/2satel_), the difference between the maximum binding energy of the main and satellite Fe 2p_3/2_ photoelectron lines (ΔBE = BE_Fe2p3/2main_ − BE_Fe2p3/2satel_) as well as the intensity ratios of the satellite and main Fe 2p_3/2_ photoelectron lines (*I_ratio_* = (*I*_Fe2p3/2satel_)/(*I*_Fe2p3/2main_)) and the sum of β-diketonato ligand Gordy scale R-group electronegativities, Σχ_R_ (Figure 8) fit the equations:

BE_Fe2p3/2satel_ = 0.1678 Σχ_R_ + 703.45; R^2^ = 0.9885 (6)

ΔBE = −0.0899 Σχ_R_ + 3.4344; R^2^ = 0.9456 (7)

*I_ratio_* = 0.0568 Σχ_R_ − 0.5505; R^2^ = 0.9794 (8)

## 3. Methods

### 3.1. Compounds

The ferrocenyl-functionalized β-diketonato manganese(III) complexes of general formula [Mn(FcCOCHCOR)_3_], with R = CF_3_, **1**, CH_3_, **2**, C_6_H_5_, **3** and Fc, **4**, as well as [Mn(CH_3_COCHCOCH_3_)_3_], **5**, and [Mn(FcCOCHCOFc)_2_(FcCOCHCOCH_3_)], **6**, (Figure 1) were prepared and characterized according to published methods [4].

### 3.2. X-Ray Photoelectron Spectroscopy

XPS data was recorded on a PHI 5000 Versaprobe (Ulvac-Phi, Chigasaki, Japan) system, with a monochromatic Al Kα X-ray source. Spectra were obtained using the aluminium anode (Al Kα = 1486.6 eV), operating at 50 μm, 12.5 W, and 15 kV energy (97 X-ray beam). A low energy neutralizer electron gun was used to minimize charging of the samples. The instrument work function was calibrated to give a binding energy of 284.5 eV for the lowest binding energy peak of the carbon 1s envelope, corresponding to adventitious carbon. Survey scans were recorded at constant pass energy of 187.85 eV, while detailed region scans were recorded at constant pass energy of 29.35 eV for C and O, and 93.90 eV for Fe and Mn. The resolution of the PHI 5000 Versaprobe system is FWHM = 0.53 eV at a pass energy of 23.5 and FWHM = 1.44 eV at a pass energy of 93.90. The background pressure was 2 × 10^−8^ mbar. Spectra have been charge-corrected to the main line of the carbon 1s spectrum, which was set to 284.5 eV. XPS data was analyzed utilizing Multipak version 8.2c computer software [49] and applying Gaussian/Lorentz fits (the Gaussian/Lorentz ratios were always > 95%). All measured photoelectron lines were seven-point smoothed.

## 4. Concluding Remarks

The sub-structures of the Mn 2p and Fe 2p XPS photoelectron lines gave insight into the electronic structure of the [Mn(β-diketonato)_3_] complexes **1**–**6**. Despite the absence of fine structure, the Mn 2p_3/2_ envelope could successfully be described with the calculated multiplet splitting from Gupta and Sen [8,9]. The Mn 2p_3/2_ envelopes of complexes containing unsymmetrically substituted β-diketonato ligands could be approximated by two sets of calculated multiplet splitting peaks corresponding to the presence of *mer* and *fac* isomers. The shake-up peak in the Mn 2p and the shake-down peak in the Fe 2p region gave a clear indication of the charge transfer process taking place from the ligand to the centrally coordinated Mn^III^ cation. A key factor that influences all these processes and binding energies is the theoretical concept “sum of group electronegativities”, Σχ_R_, of the β-diketonato pendant side groups. Utilizing the experimental XPS binding energies and Σχ_R_ values, it was shown that the *mer* isomer is more stable than the *fac* isomer but more electron-donating ligands stabilize *fac* isomers more than *mer* isomers. It also became possible to explain the intuitive feeling that ligands which are stronger electron-withdrawing induces a lower degree of covalence in bonds than ligands which are stronger electron-donating and that the latter transfer more charge to metal centers to which they are coordinated than electron-withdrawing ligands. Furthermore, it also explains the decrease in binding energy of core electrons of metals when relative stronger electron-donating ligands are coordinated to it. Variations in the XPS spectral characteristics of the Mn and Fe 2p regions as a result of changing R-group substituted on the β-diketonato ligands, (FcCOCHCOR)^−^, as well as their influence on the electronic environment of the central coordinated high spin Mn^III^ cation, is emphasized by the linear relationships involving on the one hand Σχ_R_ and on the other, binding energies of the satellite and main Fe and Mn 2p_3/2_ phototelectron lines of **1**–**4** and **6**, the difference between the maximum binding energy of the main and satellite Fe and Mn 2p_3/2_ photoelectron lines or the intensity ratios between satellite and main Fe or Mn 2p_3/2_ photoelectron lines.

## Figures and Tables

**Table 1:** χ_R_ and Σχ_R_ values, maximum binding energies, BE, of the main Mn 2p_3/2_ envelope, full width at half maximum (FWHM) values, peak separations between main Mn 2p_3/2_ and 2p_1/2_ peaks (ΔBE_1_), peak separations between satellite and Mn 2p_3/2_ peaks (ΔBE_2_), *I_ratio_* values, *mer* and *fac* isomer maximum binding energies of the main Mn 2p_3/2_ simulated peaks of **1**–**6**.

Comp. No.; Ligand R-Groups	χ_R_	Σχ_R_^4^	BE Mn 2p_3/2_ (eV)	FWHM (eV)	ΔBE_1_^5^ (eV)	ΔBE_2_^6^ (eV)	*I*_ratio_^7^	BE Mn 2p_3/2_ (eV)	
								*mer*	*fac*
**1**; CF_3_^1^	3.01	14.64	641.86	4.64	11.96	3.98	0.13	641.62	642.22
**2**; CH_3_^1^	2.34	12.63	641.53	4.00	11.79	4.42	0.19	641.5	641.9
**3**; C_6_H_5_^1^	2.21	12.24	641.50	3.86	11.76	4.51	0.18	641.38	641.68
**4**; Fc ^1^	1.87	11.22	641.31	3.86	11.58	4.74	0.23	- ^8^	- ^6^
**5**; CH_3_,CH_3_^2^	2.34	14.04	641.73	4.06	11.91	4.36	0.17	- ^8^	- ^8^
**6**; Fc,Fc,CH_3_^3^	-	11.69	641.41	4.3	11.67	4.64	0.23	- ^9^	- ^9^

^1^ For [Mn(FcCOCHCOR)_3_], **1**–**4**; ^2^ For [Mn(CH_3_COCHCOCH_3_)_3_], **5**; ^3^ For [Mn(FcCOCHCOFc)_2_(FcCOCHCOCH_3_)], **6**; ^4^ Sum of R-group electronegativities, Σχ_R_. By way of example, for **6**, this is calculated as follows: Σχ_R_ = 5(χ_Fc_) + χ_CH3_ = 5(1.87) + 2.34 = 11.69; ^5^ ΔBE_1_ = BE_Mn2p1/2_ − BE_Mn2p3/2_; ^6^ ΔBE_2_ = BE_Mn2p3/2satel_ − BE_Mn2p3/2main_; ^7^ *I_ratio_* = ratio between the intensities of the satellite and main Mn 2p_3/2_ photoelectron lines (= (*I*_Mn2p3/2satel_)/(*I*_Mn2p3/2main_)); ^8^ Symmetrical ligands implying there are no *mer* and *fac* isomers; ^9^ The ligands of complex **6** are not equivalent, hence the concepts *mer* and *fac* isomers are not applicable.

**Table 2:** Maximum binding energy, BE, of the Mn 2p_3/2_ envelope, the binding energies of each multiplet split peak and *I_ratio_* %’s for the multiplet splitting of the Mn 2p_3/2_ peak. BE of the shake-up (charge transfer peak) is also shown.

Comp. No.; R-Groups	Max BE (eV)	Isomer	Peak ^1^ (eV)	*I_ratio_* % ^7^	Peak ^2^ (eV)	*I_ratio_* % ^7^	Peak ^3^ (eV)	*I_ratio_* % ^7^	Peak ^4^ (eV)	*I_ratio_* % ^7^	Peak ^5^ (eV)	*I_ratio_* % ^7^	Shake-up (eV)	*I_ratio_* % ^7^
**1**; CF_3_^1^	641.86	*mer*	640.10	23.4	641.22	23.4	642.52	31.2	644.17	15.0	645.56	7.0	646.90	2.46
		*fac*	640.70	23.4	641.82	23.4	643.12	31.2	644.77	15.0	646.16	7.0		
**2**; CH_3_^1^	641.53	*mer*	639.93	23.1	640.99	23.1	641.83	31.4	642.93	15.3	643.71	7.1	645.77	4.56
		*fac*	640.33	23.1	641.39	23.1	642.23	31.4	643.33	15.3	644.11	7.1		
**3**; C_6_H_5_^1^	641.50	*mer*	639.86	23.0	640.90	23.0	641.82	31.0	642.90	16.0	644.03	7.0	645.71	9.28
		*fac*	640.16	23.0	641.20	23.0	642.12	31.0	643.20	16.0	644.33	7.0		
**4**; Fc ^1^	641.31	- ^5^	639.78	22.5	640.80	22.5	641.56	30.3	642.86	18.0	643.87	6.7	645.89	10.25
**5**; CH_3_,CH_3_^2^	641.73	- ^5^	640.00	22.9	641.14	22.9	642.20	31.0	643.36	16.1	644.36	7.1	646.24	3.98
**6**; Fc,Fc,CH_3_^3^	641.41	- ^6^	639.84	21.5	640.89	21.5	641.82	26	643.19	21.5	644.10	11.2	645.35	8.69
Mn^3+^ ^4^		-	640.1	23	641.4	23	642.3	31	643.1	16	644.9	7		

^1^ For [Mn(FcCOCHCOR)_3_], **1**–**4**. ^2^ For [Mn(CH_3_COCHCOCH_3_)_3_], **5**. ^3^ For [Mn(FcCOCHCOFc)_2_(FcCOCHCOCH_3_)], **6**. ^4^ Gupta and Sen calculations of the Mn(III) free ion [8,9]. ^5^ Symmetrical ligands implying there are no *mer* and *fac* isomers. ^6^ The ligands of complex **6** are not equivalent, hence the concepts *mer* and *fac* isomers are not applicable. ^7^ *I_ratio_* percentages = percentage ratio between the intensities of the satellite and main Mn 2p_3/2_ photoelectron lines (= (*I*_Mn2p3/2satel_)/(*I*_Mn2p3/2main_) × 100).

**Table 3:** Σχ_R_ values, maximum binding energy (BE) of the main and satellite Fe 2p_3/2_ envelope, peak separations between the satellite and main Fe 2p_3/2_ peaks ΔBE, as well as *I_ratio_* values for **1**–**4** and **6**.

Comp. Num.; R-Groups	Σχ_R_^3^	BE_Fe2p3/2main_ (eV)	FWHM (eV)	BE_Fe2p3/2satel_ (eV)	FWHM (eV)	ΔBE ^5^ (eV)	*I*_ratio_^f^
**1**; R = CF_3_^1^	14.64	708.03	2.67	705.91	- ^4^	2.12	0.28
**2**; R = CH_3_^1^	12.63	707.87	2.22	705.61	2.37	2.26	0.16
**3**; R = C_6_H_5_^1^	12.24	707.84	1.93	705.48	2.07	2.36	0.16
**4**; R = Fc ^1^	11.22	707.77	2.81	705.28	2.40	2.34	0.09
**6**; Fc,Fc,CH_3_^2^	11.69	707.79	1.95	705.45	1.95	2.49	0.11

^1^ For [Mn(FcCOCHCOR)_3_], **1**–**4**. ^2^ For [Mn(FcCOCHCOFc)_2_(FcCOCHCOCH_3_)], **6**. ^3^ Sum of R-group electronegativities; calculations are explained in Table 1. ^4^ Due to a non-linear baseline, FWHM of **1** could not be measured with any degree of accuracy, but it is estimated as 2.84 eV. ^5^ ΔBE = BE_Fe2p3/2main_ − BE_Fe2p3/2satel_. ^f^ *I_ratio_* = ratio between the intensities of the satellite and main Fe 2p_3/2_ photoelectron lines (= (*I*_Fe2p3/2satel_)/(*I*_Fe2p3/2main_)).
